# Valorisation of hydrothermal liquefaction wastewater in agriculture: effects on tobacco plants and rhizosphere microbiota

**DOI:** 10.3389/fpls.2023.1180061

**Published:** 2023-06-05

**Authors:** Wanda Gugliucci, Valerio Cirillo, Albino Maggio, Ida Romano, Valeria Ventorino, Olimpia Pepe

**Affiliations:** ^1^ Department of Agricultural Sciences, Division of Microbiology, University of Naples Federico II, Naples, Italy; ^2^ Department of Agricultural Sciences, Division of Plant Biology and Crop Science, University of Naples Federico II, Naples, Italy; ^3^ Task Force on Microbiome Studies, University of Naples Federico II, Naples, Italy

**Keywords:** HTL wastewater, microbiota, high-throughput sequencing, microbial community, biodiversity, *Nicotiana tabacum L.*

## Abstract

Industrial wastewater obtained from hydrothermal liquefaction (HTL-WW) of food wastes for biofuels production could represent a source of crop nutrients since it is characterized by a high amount of organic and inorganic compounds. In the present work, the potential use of HTL-WW as irrigation water for industrial crops was investigated. The composition of the HTL-WW was rich in nitrogen, phosphorus, and potassium with high level of organic carbon. A pot experiment with *Nicotiana tabacum* L. plants was conducted using diluted wastewater to reduce the concentration of some chemical elements below the official accepted threshold values. Plants were grown in the greenhouse under controlled conditions for 21 days and irrigated with diluted HTL-WW every 24 hours. Soils and plants were sampled every seven days to evaluate, over time, the effect of wastewater irrigation both on soil microbial populations, through high-throughput sequencing, and plant growth parameters, through the measurement of different biometric indices. Metagenomic results highlighted that, in the HTL-WW treated rhizosphere, the microbial populations shifted *via* their mechanisms of adaptation to the new environmental conditions, establishing a new balance among bacterial and fungal communities. Identification of microbial taxa occurring in the rhizosphere of tobacco plants during the experiment highlighted that the HTL-WW application improved the growth of Micrococcaceae, Nocardiaceae and Nectriaceae, which included key species for denitrification, organic compounds degradation and plant growth promotion. As a result, irrigation with HTL-WW improved the overall performance of tobacco plants which showed higher leaf greenness and increased number of flowers compared to irrigated control plants. Overall, these results demonstrate the potential feasibility of using of HTL-WW in irrigated agriculture.

## 1 Introduction

The competition for fresh water among industry, agriculture, and public utilities is increasing due to population growth and climate change (Zucchinelli et al., 2021). On average, 40% of total water abstraction in Europe is used for industry and energy production, 15% for public water supply and 44% for agriculture. Agriculture is therefore the major user of freshwater. At the same time, agriculture is the most affected sector by water scarcity, especially in Mediterranean countries due to an aggravation of semi-arid and arid climatic conditions (Sofroniou and Bishop, 2014). In this scenario, the key to ensure water security is “water reuse”, also commonly known as water recycling or water reclamation. Reclaimed water for irrigation includes different sources such as municipal and industrial wastewater, stormwater, agriculture runoff and return flows (EPA, 2021). About 380 billion m^3^ of water can be recovered from the annual volumes of wastewater produced. Water recovery is expected to reach 470 billion m^3^ by 2030 and 574 billion m^3^ by 2050 (United Nations Educational, Scientific and Cultural Organization – UNESCO, 2021). The use of wastewater in agriculture offers several benefits including the availability of extra sources to cope with water scarcity; the possibility of reserving better-quality water for human consumption; the recovery of the nutrients contained in wastewater; replenishment of soil organic matter; reduction of wastewater release into the surrounding area; limiting/avoiding marine intrusion in coastal areas and overexploitation of fresh water (Ungureanu et al., 2020). Wastewater recycling not only offers an alternative source for crop irrigation, but also the opportunity to recover fertilizing elements, such as nitrogen (N), phosphorous (P), potassium (K), organic matter, minerals, and micronutrients. Nevertheless, according to Petousi et al. (2019), in Europe only the 2.4% accounting for 700 Mm^3^/year of wastewater is reused and mostly in Spain. Until 2020, the major barriers preventing a wider spreading of this practice in EU were the limited awareness of potential benefits among stakeholders, and the lack of a supportive and coherent framework for water reuse. According to Directive 91/271/EEC - Article 12, “*treated wastewater must be reused whenever appropriate and disposal routes must minimize any adverse effects on the environment*”. However, the document does not specify the minimum quality requirements for wastewater reuse (Petousi et al., 2019). Recently, the EU commission approved a new regulation on minimum requirements for water reuse in agricultural irrigation (EU 2020/741) that will be applied starting from 26 June 2023. This will eventually encourage and facilitate water reuse across Europe.

In Italy, the agricultural use of wastewater is currently regulated by Ministerial Decree no.185/2003 (limits for heavy metals in mg/L: Al 13, As 0.02, Ban 10, Be 0.1, B 1, Cd 0.005, Co 0.05, Cr 0.1, Fe 22, Mn 0.22, Hg 0.001, Ni 0.2, Pb 0.1, Cu 1, Se 0.01, Ti 0.001, V 0.1, Zn 0.5, Sn 3; limits for macro and micronutrients: BOD, 20 mg O_2_/L; COD, 100 mg O_2_/L; Total Nitrogen, 15 mg N/L; Ammonia Nitrogen, 5 mg NH_4_/L; Total Phosphorous, 2 mg P/L; Sulphates, 500 mg SO_4_/L; Chlorides, 100 mg Cl/L), which regards only the municipal and agro-industrial effluents. Most research on testing the use of wastewater in agriculture refers to treated municipal wastewater, olive mill wastewater, sewage sludges and digestates (Food and Agriculture Organization – FAO, 2022). However, there are additional industrial processes which deliver, as side products, high levels of liquid wastes that could be recovered and valorised for agricultural uses. In particular, the hydrothermal liquefaction (HTL) is a high-performance and eco-sustainable thermochemical technology to produce bioenergy from organic biomass and wastes. According to UNEP Food Waste Index report, 2021 (UNEP Food Waste Index report, 2021), a total of around 931 million tonnes of food waste from households, food service and retail were produced worldwide in 2019. In undeveloped countries more than 90% of the waste is openly dumped, burned, or landfilled without landfill gas collection systems, resulting in high GHG emissions. The available technologies for organic waste disposal and management include landfilling, incineration, composting, and anaerobic digestion. Hydrothermal liquefaction is an additional option, especially suitable for feedstock with high moisture content, such as the sorted organic fraction of household waste. Typically, HTL is carried out in the absence of oxygen, at temperature ranging between 250 and 350°C, under autogenic pressure (50–200 bar) and 15–90 minutes reaction times. The main steps of this reaction are: (i) depolymerization of macromolecules to form water soluble monomers, (ii) dehydration, decarboxylation, and deamination to degrade the different monomers, (iii) recombination of the reactive fragments to form a water insoluble bio-oil, (iv) further polymerization at prolonged reaction time to form hydro-char. At the end of this process, four main outputs are produced: the water insoluble bio-oil, the water phase containing organic and inorganic components, the gaseous phase, mainly constituted by CO_2_ and the solid residues consisting in hydro-char and ash (Gu et al., 2019). The bio-oil, which can be used directly as low sulphur fuel or further refined to produce high-performance biofuels. Depending on the moisture content of the starting feedstock, the process generates also up to 95% of hydrothermal liquid wastewater (HTL-WW) with high concentrations of organic and inorganic compounds/elements. Therefore, the valorisation of this liquid co-product is a crucial step in hydrothermal liquefaction development since its discharge into civil wastewater treatment plants requires high extra-costs, making this process no longer economic viable (Posmanik et al., 2017). Moreover, HTL-WW may have some interesting properties as irrigation water, since it is rich in plant macro and micronutrients as well as organic carbon. The HTL-WW does not contain pathogens, pesticides and emerging contaminants including analgesics, antihypertensive drugs favouring its reuse in different areas (Jaramillo and Restrepo, 2017). Nevertheless, the presence of organic and inorganic matter in wastewaters could affect the soil physic-chemical properties including the electrical conductivity (EC), hydrophobicity, heavy‐metal concentrations, pH as well as organic carbon content, humus, nitrogen, phosphate, and potassium levels and should be adequately monitored (Muamar et al., 2014). Moreover, wastewater applications are expected to alter the soil microbiota, because it is particularly sensitive to human-induced perturbations or environmental stress compared to higher organisms due to their close relations with the surroundings and because of higher surface area to volume ratio (Karimi et al., 2017). Investigating the soil microbiota composition and the interactions with plant systems could provide useful information on both crops and soils productivity and health status (Ventorino et al., 2018a). In this context, the aim of this study was to assess the feasibility of the use of HTL-WW as irrigation water in agriculture using *Nicotiana tabacum* L. as a model plant since this species is easy to grow and does not have problems in terms of pathogens, allowing to reduce the interactions with other environmental constraints and have a cleaner assessment of the effects of HTL-WW on plant growth. Moreover, the impact of HTL-WW on root-associated microbiota was also determined and described by evaluating diversity and richness variations of bacterial and fungal communities. To the best of our knowledge, this is the first work reporting the use of wastewater deriving from hydrothermal liquefaction for crop irrigation purpose.

## 2 Material and methods

### 2.1 HTL-WW analysis

The HTL-WW used in this study was provided by the Eni Renewable Energy and Material Science Research Center (Novara, Italy). Two samples were taken from the Waste to Fuel HTL pilot plant in Eni Gela Refinery (Caltanissetta, Italy) at different time. For each sample, two 1 L aliquots were taken from two different storage tanks and were analysed to quantify their chemical composition. The HTL-WW was, on average, acidic with a pH value of 4.75 and Chemical Oxygen Demand (COD) and Total Organic Carbon (TOC) of 57250 and 22433.5 mg/L, respectively. It contained considerable amounts of total nitrogen (TN) (1845.75 mg/L) including ammonia (710 mg/L) and nitrates (9.8 mg/L), phosphate (24.45 mg/L), sulphate (23.7 mg/L) and potassium (3130 mg/L). The concentrations of macronutrients were: 50 mg/L of Ca, 25 mg/L of Mg, 23.5 mg/L of S. The heavy metals concentration (mg/kg) was composed by 0.3 of Cu, 8 of Fe, 2.9 of Zn, 0.1 of Ni, 6 of Al, 0.4 of Cr and 0.1 of Mo. The electrical conductivity reached 12.9 mS/cm, directly related to the high concentrations of Na^+^ and Cl^-^, 2990 and 1784 mg/L, respectively.

The HTL-WW was diluted 1:10 with tap water to reduce the values of some parameters below the threshold limits reported in the Ministerial Decree n.185/2003, which regulates the agricultural use of wastewater (**Table 1**).

**Table 1:** Comparison between the main parameters of HTL-WW from the *waste to fuel* process and the legislative limits (DM 2003/185) for wastewater use in agriculture.

	HTL-WW	Diluted HTL-WW (1:10)	Italian thresholds (DM183/2003)
**pH**	5	5	5-9.5
**TSS**	29.5 mg/L	2.95 mg/L	10 mg/L
**COD**	57250 mg/L	5725 mg/L	100 mg/L
**NH_3_**	710 mg/L	71 mg/L	2 mg/L
**E.C.**	12900 mS/cm	1290 mS/cm	3000 mS/cm

TSS, Total Suspended Solids; COD, Chemical Oxygen Demand; NH_3_, ammonia; E.C., Electrical Conductivity.

### 2.2 Experimental design and plant growth conditions

The tobacco experiment was set up in a greenhouse located at the experimental station of the University of Naples Federico II, Southern Italy (lat. 43°31’N, long. 14°58′E; alt. 60 m above sea level) with *Nicotiana tabacum* L. plants. The germination of tobacco seeds was carried out in peats under controlled conditions (T 28°C, 16 h light/8h, Ur ~ 60%) until the appearance of the 3rd-4th true leaf. Plants were transplanted in 19 cm Ø plastic pots and drip irrigated with nutrient solutions (N-P-K 20:20:20). At 60 Days-After-Sowing (DAS), when plants were at the 4-5 true leaves, half of the plants were irrigated with HTL-WW diluted in water at a ratio of 1:10, while control plants were irrigated with tap water. Irrigation was performed for 21 days.

### 2.3 Soil and rhizosphere sampling

Soil samples were collected after irrigation with HTL-WW from each pot at the beginning of the experiment (T0) and at 7 (T7), 14 (T14 and 21 (T21) days. For the microbiological analysis tobacco rhizosphere were sampled according to Romano et al. (2020a). The soil chemical parameters were analysed before and after treatment. In particular, after soil drying (65°C) were determined the pH-H_2_O (1:2.5 soil:water solution ratio – pH meter GLP 22, Crison, Barcelona, Spain); electrical conductivity (E.C.) (1:5 soil:water solution ratio—Conductimeter basic 30, Crison, Barcelona, Spain); total nitrogen (N) concentration, assessed after acid digestion with sulfuric acid (96%) in the presence of potassium sulphate and a low concentration of copper by the Kjeldahl method (Kjeldahl, 1883); total carbon (TC), measured according to the Walkley–Black method and reported as soil organic matter (SOM% = C% × 1.726); phosphorus (P_2_O_5_) content, following the Olsen method and finally the ammonium acetate method was adopted for exchangeable potassium (K_2_O) measurement.

### 2.4 Microbiological analysis

Microbiological analysis was performed on fresh soil samples. For molecular analysis, total genomic DNA was extracted from tobacco rhizosphere using a FastDNA SPIN Kit for Soil (MP Biomedicals, Illkirch Cedex, France) according to the manufacturer’s instructions.

#### 2.4.1 High-throughput sequencing

Synthetic oligonucleotide primers S-D-Bact-0341F50 (5′-CCTACGGGNGGCWGCAG-3′) and S-D-Bact-0785R50 (5′-GACTACHVGGGTATCTAATCC-3′) (Klindworth et al., 2013) and the primers EMP.ITS1 (5′-CTTGGTCATTTAGAGGAAGTAA-3′) and EMP.ITS2 (5′-GCTGCGTTCTTCATCGATGC-3′) (Bokulich and Mills, 2013) were used to evaluate bacterial and fungal diversity, respectively, by amplicon-based metagenomic sequencing. PCR conditions for V3-V4 region consisted of 25 cycles (95°C for 30 s, 55°C for 30 s and 72°C for 30 s) plus one additional cycle at 72°C for 10 min as a final chain elongation. PCR conditions for ITS1-ITS2 region consisted of 35 cycles (94°C for 30 s, 52°C for 30 s and 68°C for 30 s) plus one additional cycle at 68°C for 7 min as a final chain elongation. Agencourt AMPure beads (Beckman Coulter, Milan, IT) were used to purify PCR products; whereas, quantification was performed by AF2200 Plate Reader (Eppendorf, Milan, IT). Equimolar pools were obtained, and sequencing was carried out on an Illumina MiSeq platform, yielding to 2× 250 bp, paired end reads.

#### 2.4.2 Bioinformatics and data analysis

After sequencing, QIIME 2 software was used to analyse fastq files (Bolyen et al., 2019). Sequence adapters and primers were trimmed by using cut adapter, whereas DADA2 algorithm (Callahan et al., 2016) was used to trim low quality reads, to remove chimeric sequences, and joined sequences shorter than 250 bp by using the DADA2 denoise paired plugin of QIIME2. DADA2 produced amplicon sequence variants (ASVs), which were rarefied at the lowest number sequences/sample and used for taxonomic assignment using the QIIME feature-classifier plugin against Greengenes and UNITE database for the bacterial and fungal microbiota, respectively. The taxa abundances were recalculated after the exclusion of chloroplast, mitochondria contaminants and singleton ASVs.

Alpha diversity metrics (Shannon index, Faith’s PD index and Observed_OTUs) was computed by QIIME2 and the pairwise Kruskal-Wallis’ test was used for significant differences. Beta diversity was also calculated and plotted by QIIME 2 for the Weighted UniFrac (Lozupone et al., 2006).

Bar-plots were generated using R packages phyloseq 1.38.0 (McMurdie and Holmes, 2013) and ggplot2 3.4.2. Furthermore, the metabolic function was predicted by Tax4Fun analysis through Kyoto Encyclopedia of Genes and Genomes (KEGG) database (Wang et al., 2019). Analysis mainly focused on the differences of predicted abundances of genes involved in nitrogen fixation, 1-aminocyclopropane-1-carboxylate (ACC) deaminase, aminopeptidase, and glucanase activities across treated with HTL-WW and untreated samples. Heatmaps were generated in R using the package Pheatmap 1.0.12 (Kolde, 2019).

### 2.5 Biometric and physiological measurements

At 7, 14 21 days after HTL-WW treatment (DAT), three plants per treatments have been sampled for shoot biomass determination. At the same time points, SPAD index was measured with a MINOLTA chlorophyll meter (SPAD 502-Plus). The flower biomass was measured at the end of the experiment (after 21 days).

Data were analysed by test-t for pairwise comparison of means (at P < 0.05) using SPSS 19.0 statistical software package (SPSS Inc., Cary, NC, United States).

## 3 Results

### 3.1 Phenotypic and physiological evaluation of *Nicotiana tabacum L* plants

**Figure 1:**
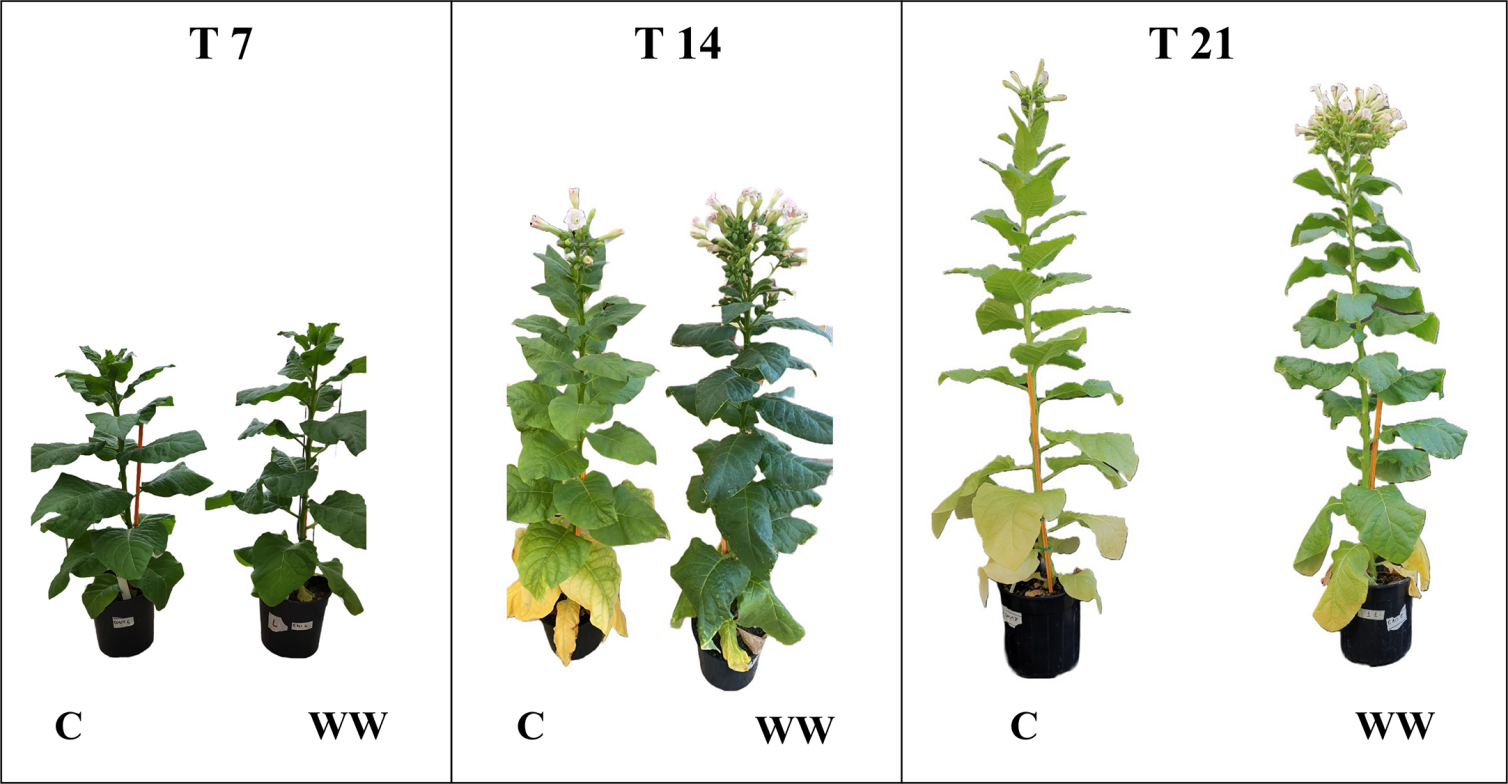
*Nicotiana tabacum* L. plants treated (WT) and untreated (C) with HTL-WW deriving from *Waste to Fuel* industrial process after 7 (T7), 14 (T14) and 21 (T21) days of growth.

**Figure 2:**
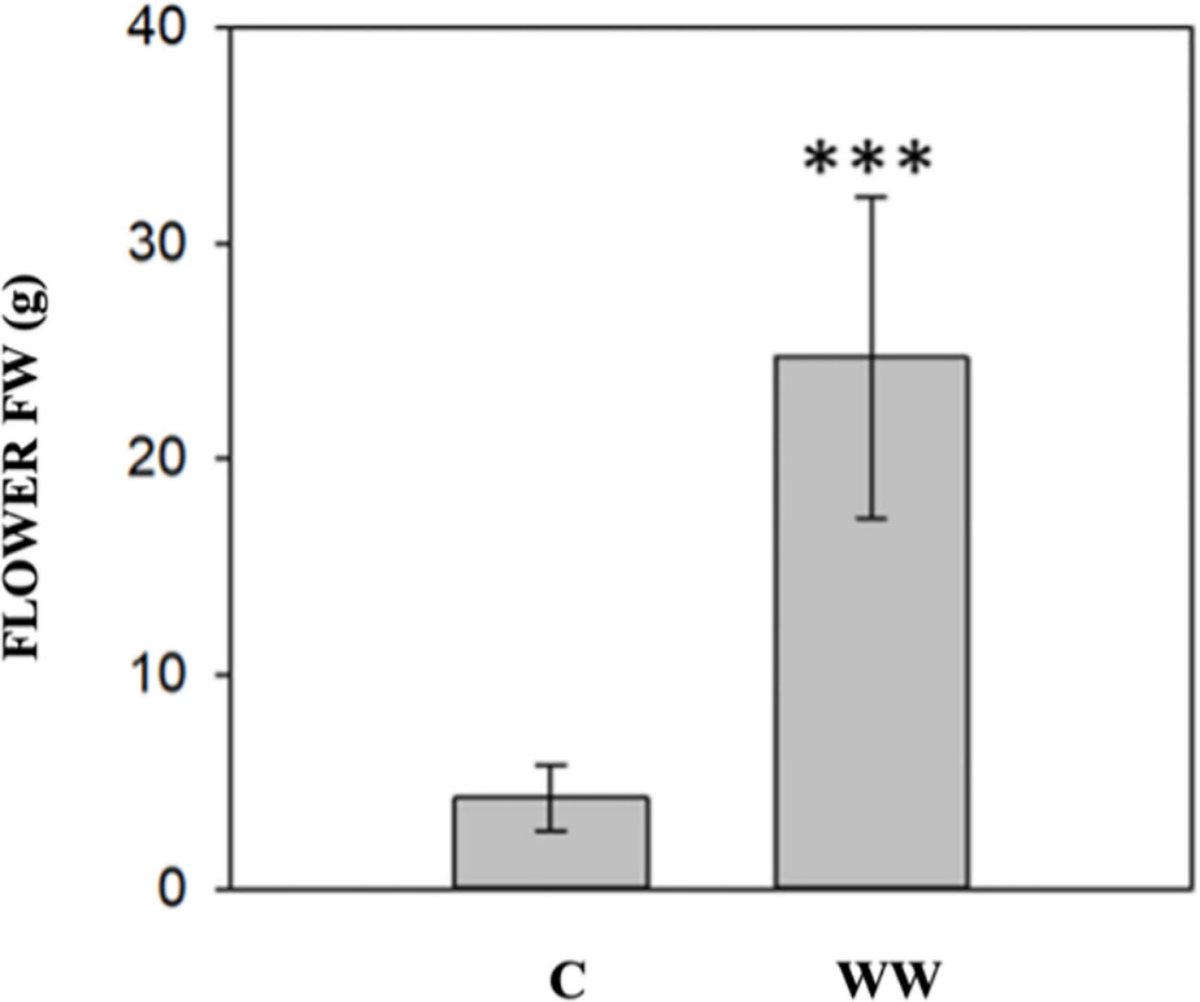
Flower fresh weight (FW) of *Nicotiana tabacum* irrigated with tap water (C) and with HTL-WW (WW) at the end of the experiment (21 days). Test-t (p<0.05) was performed to identify significant differences between the treatments (*** = p<0.001).

The impact of HTL-WW irrigation on *Nicotiana tabacum* L. plants was evaluated by phenotypic observations and biometric measurements carried out every seven days. Phenotype differences between treated and untreated plants increased with the time of growth. After 14 and 21 days, plants treated with HTL-WW showed a more intense tissue pigmentation (**Figure 1**) and higher SPAD values (T14 + 13.6% and T21 + 46.8%) (**Table 2**). Despite the greener/healthier appearance of wastewater treated plants, no significant differences were found in the fresh weight of the shoot biomass between treated (HTL-WW) and untreated (C) plants (**Table 3**). The flower biomass at the end of the experiment (T21) was significantly higher (T-test: 0.007; P <0.05) in plants irrigated with HTL-WW (34.05 ± 14 g) compared to untreated plants (6.25 ± 2.67 g) (**Figure 2**). The main chemical properties of the soils collected from the tobacco’s pots before (T0) HTL-WW treatment were total nitrogen (TN) 0.18%, electrical conductivity (EC) 420.0 μS/cm, organic matter (OM) 6.07%, phosphate (P_2_O_5_) 223.514 ppm, potassium (K_2_O) 994.8 ppm and pH 7.3, remaining constant after the experiment.

**Table 2:** SPAD index of *Nicotiana tabacum* treated (HTL-WW) and untreated (C) with HTL-WW after 7 (T7), 14 (T14) and 21 (T21) days of growth.

	SPAD		
Treatment	T7	T14	T21
**Control**	43.3 ± 0.95	37.2 ± 0.68	31.4 ± 0.56
**HTL-WW**	42.9 ± 0.85	41.9 ± 0.75	46.1 ± 0.95
**Test-t**	*ns*	***	***

Test-t (p<0.05) was performed to identify significant differences between the treatments (ns, not significant; *** = p<0.001).

**Table 3:** Fresh biomass accumulation (shoot FW) of *Nicotiana tabacum* treated (HTL-WW) and untreated (C) with HTL-WW after 7 (T7), 14 (T14) and 21 (T21) days of growth.

		Shoot FW (g)	
Treatment	T7	T14	T21
**Control**	145.3 ± 13.39	183.5 ± 6.51	222.8 ± 22.36
**HTL-WW**	139.2 ± 6.11	230.0 ± 17.61	246.7 ± 23.22
**Test-t**	*ns*	*ns*	*ns*

Test-t (p<0.05) was performed to identify significant differences between the treatments (ns, not significant).

### 3.2 Alpha and beta diversity of microbial community

The microbial diversity was characterized by partial 16S rRNA gene and ITS region (ITS1, 5.8S and ITS2) sequencing obtained from DNA directly extracted from rhizosphere samples of *Nicotiana tabacum* L. plants irrigated with HTL-WW (WW) or with tap water (C). In total, 652,845 and 49,184 high quality reads were analysed for prokaryotes and eukaryotes, respectively. The alpha-diversity was determined by calculating the Shannon diversity index, Faith’s Phylogenetic Diversity index and Observed OTUs based on OTUs of 99% identity.

**Figure 3:**
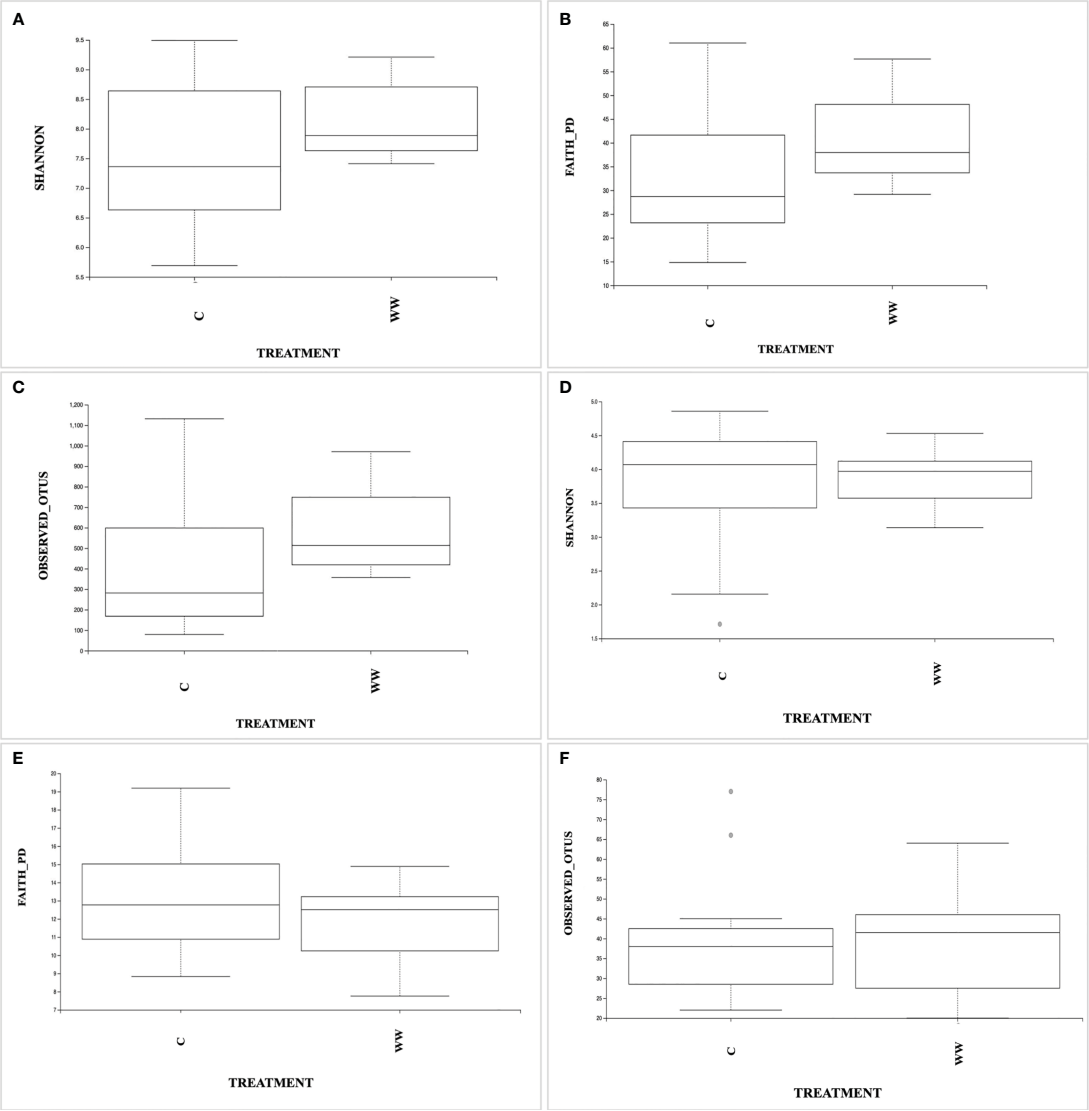
The box plots showing Shannon diversity, Faith’s Philogenetic Diversity, and Observed OTUs indices based on prokaryotic **(A–C)** and eukaryotic **(D–F)** communities in the rhizosphere samples irrigated with HTL-WW (WW) and tap water (C).

As shown in **Figure 3**, no significant differences (p > 0.05) in bacterial and fungal diversity were found between HTL-WW treated (WW) and untreated (C) tobacco rhizosphere, as revealed by the Shannon (**Figures 3A, D**), Faith’s Phylogenetic Diversity indices (**Figures 3B, E**) and Observed OTUs (**Figures 3C, F**).

**Figure 4:**
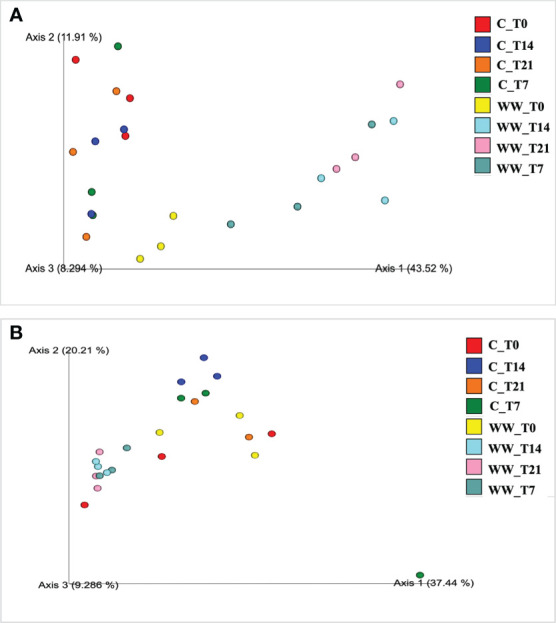
Principal Coordinates Analysis (PCoA) of weighted UniFrac distances of prokaryotic **(A)** and eukaryotic **(B)** communities of tobacco rhizosphere. WW_T0, plants irrigated with HTL-WW at time 0; WW_T7, plants irrigated with HTL-WW after 7 days; WW_T14, plants irrigated with HTL-WW after 14 days; WW_T21, plants irrigated with HTL-WW after 21 days; C_T0, plants irrigated with tap water at time 0; C_T7, plants irrigated with tap water after 7 days; C_T14, plants irrigated with tap water after 14 days; C_T21, plants irrigated with tap water after 21 days.

The PCoA of the weighted UniFrac community distance showed a marked difference between the microbiota of HTL-WW treated and untreated rhizosphere samples, highlighting that the use of industrial wastewater exerts selection pressure on the soil microbiota, especially for the bacterial communities (**Figure 4**). In fact, the sample of treated rhizosphere grouped separately on the right side of the chart in **Figure 4A** compared to untreated rhizosphere highlighting a separation of the samples based on irrigation treatment. It was interesting to note how the tobacco rhizosphere irrigated with HTL-WW at the beginning of the experiment (WW_T0) grouped with the control samples irrigated with tap water. However, the samples irrigated with the wastewater gradually separated over time from the control rhizosphere (**Figure 4A**).

A similar trend was observed for the fungal community structure, although the distribution of the samples was less marked (**Figure 4B**). In fact, the rhizosphere of the treated plants at the beginning of the experiment (WW_T0) was more grouped with the control samples irrigated with tap water; while samples irrigated with wastewater over time slightly separated among them (**Figure 4B**).

### 3.3 Microbial taxonomic composition

To evaluate any alteration in the microbial communities’ structure after the HTL-WW irrigation treatment, the relative abundances of bacterial and fungal taxa were determined at phyla and family level.

#### 3.3.1 Bacteria

**Figure 5:**
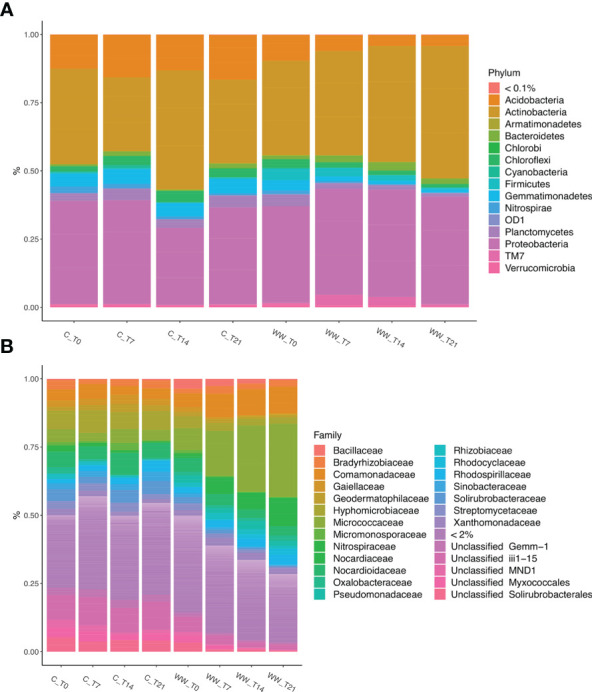
Relative abundance of bacterial phyla **(A)** and families **(B)** in the rhizosphere of *Nicotiana tabacum* L. plants treated (WW) and untreated (C) with HTL-WW. Only phyla with an abundance > 0.1% or families with an abundance > 2% in at least one sample are shown in the legend. WW_T0, plants irrigated with HTL-WW at time 0; WW_T7, plants irrigated with HTL-WW after 7 days; WW_T14, plants irrigated with HTL-WW after 14 days; WW_T21, plants irrigated with HTL-WW after 21 days; C_T0, plants irrigated with tap water at time 0; C_T7, plants irrigated with tap water after 7 days; C_T14, plants irrigated with tap water after 14 days; C_T21, plants irrigated with tap water after 21 days.

In total, fifteen different bacterial phyla were detected in the soil samples with a relative abundance > 0.1%. Proteobacteria and Actinobacteria were the taxa which heavily dominated the bacterial composition of tobacco rhizosphere. These phyla together accounted for approximately from 63% to 90% of the total bacterial biodiversity in the rhizosphere of plants treated and untreated with HTL-WW. Additionally, Acidobacteria, Armatimonadetes, Bacteroidetes, Chlorobi, Choroflexi, Cyanobacteria, Firmicutes, Gemmatimonadetes, Nitrospirae, Planctomycetes, and Verrucomicrobia, along with candidate phylum OD1 and TM7 showed a relative abundance > 0.1% in at least one sample (**Figure 5A**).

The relative frequency of Proteobacteria was enough uniform in function of the experimental sampling time in both treated and untreated plants, whereas Actinobacteria showed a different trend. In particular, the concentration of this phylum was constant in the rhizosphere of control plants irrigated with tap water (range of relative abundance: 31-34%), with a peak at day 14 (43%). Conversely, in the rhizosphere of tobacco plants treated with HTL-WW a constant increase was observed over time, from about 34% at the beginning of the experiment (WW_T0) to 48% after 21 days of treatment (WW_T21) (**Figure 5A**). The other abundant phyla showed a different trend between the samples treated and untreated with wastewater. In fact, while Chloroflexi exhibited a similar relative abundance in all the samples, Acidobacteria, Gemmatimonadetes and Planctomycetes were mainly present in the rhizosphere of the control plants irrigated with tap water; while Firmicutes, Bacteroidetes and the candidate phylum TM7 showed a higher relative abundance in the rhizosphere of plants irrigated with HTL-WW (**Figure 5A**).

The microbial diversity was also analysed at a deeper taxonomic level. In particular, the analysis of the sequences at family level, considering only those with an abundance > 2% in at least one sample, allow to identify 20 bacterial families and 5 unclassified taxa (**Figure 5B**). These taxa showed a different relative abundance depending on the time and on the type of water used for irrigation (HTL-WW or tap water). Micrococcaceae and Nocardiaceae were most abundant in the wastewater treated (WW) rhizosphere. In detail, the relative abundance of Micrococcaceae was on average about 4% in the control samples (C), while in the treated rhizospheres there was a constant increase from 8% at the beginning of the experiment (WW_T0) up to 27% after 21 days of irrigation (WW_T21; **Figure 5B**). Nocardiaceae was almost totally absent in the rhizosphere of control plants, while it showed a relative abundance of at least 4% in the rhizosphere of plants treated with HTL-WW, especially at the end of the experiment after 21 days of irrigation (WW_T21) constituting about 13-14% of the total bacterial biodiversity (**Figure 5B**). Bacillaceae and Pseudomonaceae exhibited different behaviour. In fact, although these two families were present mainly in the rhizosphere of plants treated with wastewater, they decreased over time from 3-6% at the beginning of the experiment (WW_T0) to about 0.8-0.2% at day 21 (WW_T21; **Figure 5B**). Although Comamonadaceae was recovered in all samples, its relative abundance increased over time in the wastewater treated rhizosphere. Differently, Solirubrobacteraceae, Gaiellaceae and Sinobacteraceae were more abundant in the rhizosphere of control plants, and they decreased over time in the rhizosphere of HTL-WW treated plants (**Figure 5B**).

#### 3.3.2 Fungi

**Figure 6:**
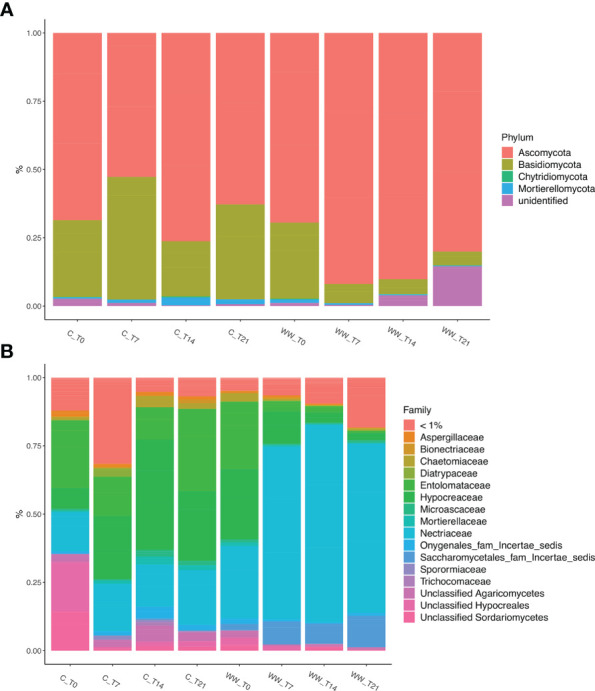
Relative abundance of fungal phyla **(A)** and families **(B)** in the rhizosphere of *Nicotiana tabacum* L. plants treated (WW) and untreated **(C)** with HTL-WW. Only families with an abundance > 1% in at least one sample are shown in the legend. WW_T0, plants irrigated with HTL-WW at time 0; WW_T7, plants irrigated with HTL-WW after 7 days; WW_T14, plants irrigated with HTL-WW after 14 days; WW_T21, plants irrigated with HTL-WW after 21 days; C_T0, plants irrigated with tap water at time 0; C_T7, plants irrigated with tap water after 7 days; C_T14, plants irrigated with tap water after 14 days; C_T21, plants irrigated with tap water after 21 days.

The phylum-level taxonomic analysis of the fungal community showed that Ascomycota and Basidiomycota dominated in all samples (WW and C), accounting for > 90% of the total biodiversity in each sample (**Figure 6A**), followed by Chytridiomycota and Mortierellomycota. Ascomycota and Basidiomycota exhibited different pattern in the treated and untreated rhizosphere. In fact, while Ascomycota was mainly present in the rhizosphere of HTL-WW treated plants, Basidiomycota showed a greater relative abundance in the rhizosphere of control plants irrigate with tap water (**Figure 6A**).

The analysis of the relative abundance of fungal families present in the tobacco rhizosphere samples with an abundance >1% in at least one sample, allowed to identify 11 fungal families (Aspergillaceae, Bionectriaceae, Chaetomiaceae Diatrypaceae, Entolomataceae, Hypocreaceae, Microascaceae, Mortierellaceae, Nectriaceae, Sporormiaceae and Trichocomaceae) which accounted for >70% of the total biodiversity, together with taxa belonging to different families without taxonomic assignment or identified only at higher taxonomic level such as Onygenales_fam_Incertae_sedis, Saccharomycetales_incertae_sedis, and unclassified Agaricomycetes, Hypocreales and Sordariomycetes (**Figure 6B**). As observed for the bacterial community, the fungal taxa were differentially present depending on the time and type of water used for irrigation. In particular, the family Nectriaceae, belonging to the Ascomycota phylum, was more abundant in the HTL-WW treated rhizosphere (WW) and increased over time. In detail, the relative abundance of Nectriaceae was on average <25% in the control samples (C), while in the treated rhizosphere there was a constant increase from about 25% at the beginning of the experiment (WW_T0) to about 53% after 21 days of irrigation (WW_T21; **Figure 6B**). On the contrary, Entolomataceae, belonging to *Basidiomycota* phylum, was more abundant in control samples, although in treated rhizo-soils was present at the beginning of experiment (WW_T0) and gradually decreased until it disappeared at day 21 (**Figure 6B**).

### 3.4 Functional prediction analysis

**Figure 7:**
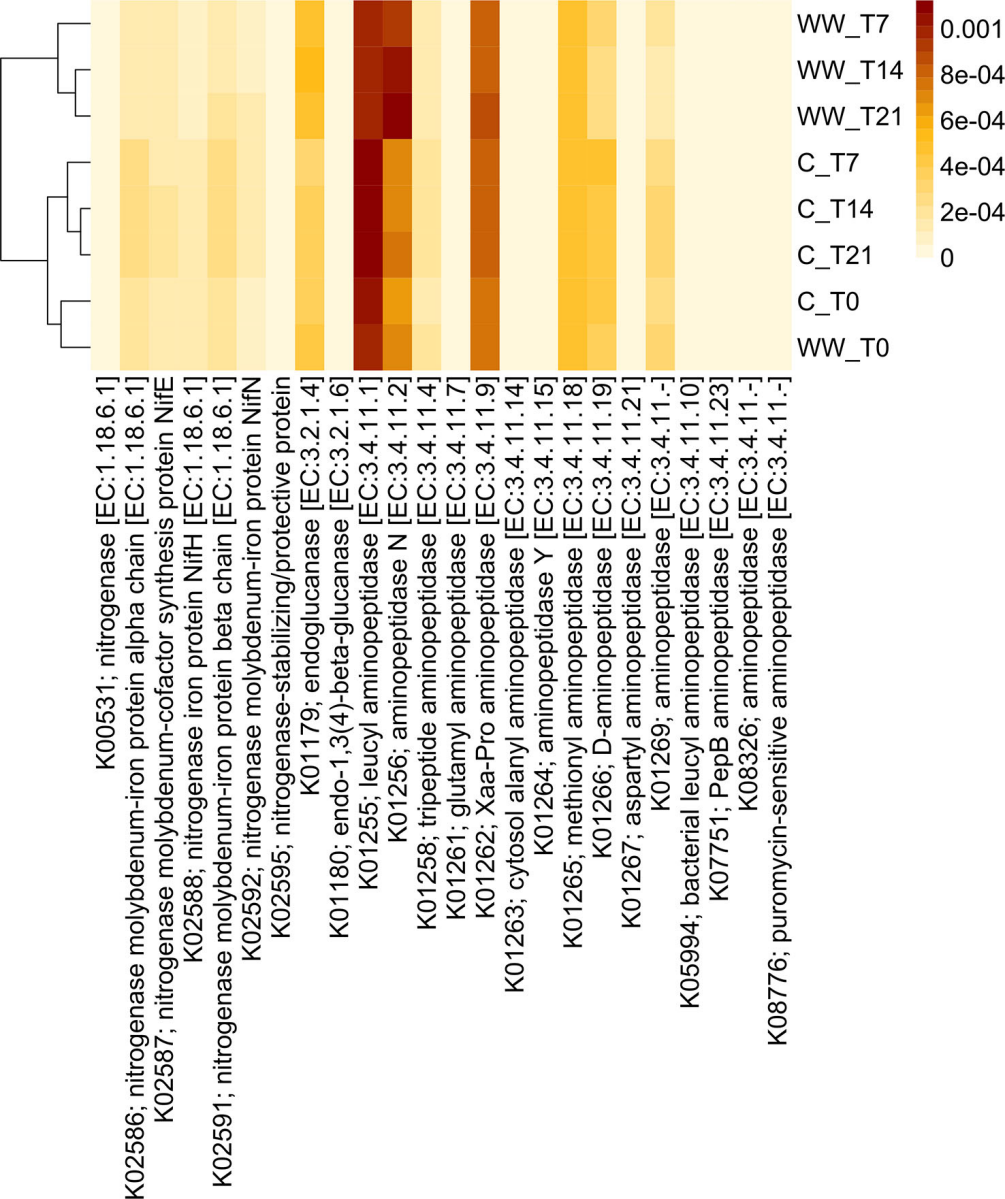
Predicted abundances of enzyme-encoding aminopeptidase, nitrogenase and glucanase genes. The colour code refers to gene abundance, with high predicted abundances (brown) and low predicted abundances (light yellow). WW_T0, plants irrigated with HTL-WW at time 0; WW_T7, plants irrigated with HTL-WW after 7 days; WW_T14, plants irrigated with HTL-WW after 14 days; WW_T21, plants irrigated with HTL-WW after 21 days; C_T0, plants irrigated with tap water at time 0; C_T7, plants irrigated with tap water after 7 days; C_T14, plants irrigated with tap water after 14 days; C_T21, plants irrigated with tap water after 21 days.

**Figure 8:**
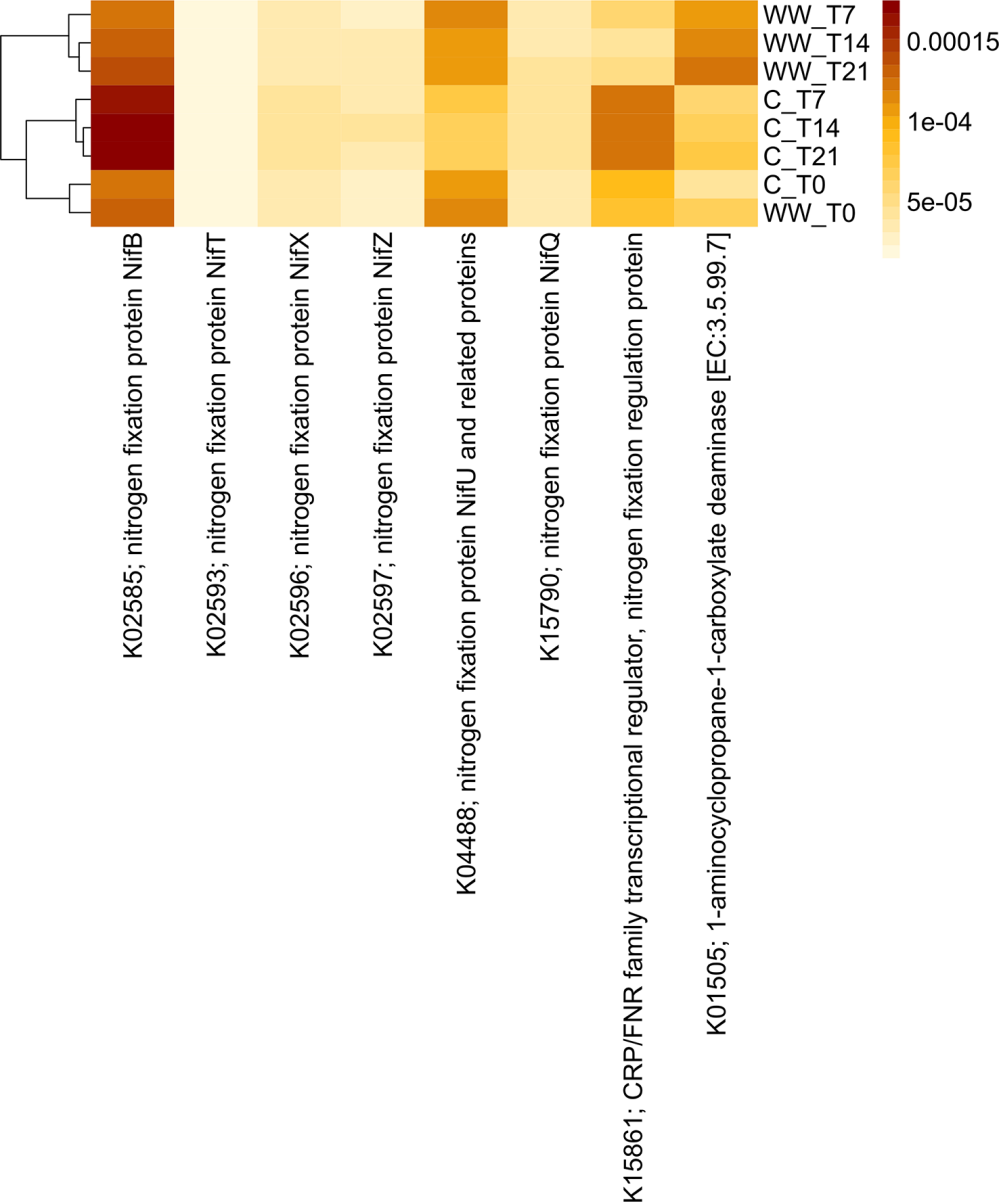
Predicted abundances of enzyme-encoding genes involved in the nitrogen fixation and ACC deaminase genes. The colour code refers to gene abundance, with high predicted abundances (brown) and low predicted abundances (light yellow). WW_T0, plants irrigated with HTL-WW at time 0; WW_T7, plants irrigated with HTL-WW after 7 days; WW_T14, plants irrigated with HTL-WW after 14 days; WW_T21, plants irrigated with HTL-WW after 21 days; C_T0, plants irrigated with tap water at time 0; C_T7, plants irrigated with tap water after 7 days; C_T14, plants irrigated with tap water after 14 days; C_T21, plants irrigated with tap water after 21 days.

Functional profiles were predicted based on the 16S rRNA gene sequencing data to assess differences between HTL-WW treated and non-treated tobacco rhizosphere. Although this analysis allowed us to analyse over 6000 functional genes, predicted abundances of some interesting enzyme‐encoding genes associated with plant growth-promoting traits and soil fertility were reported (**Figures 7**, **8**). In detail, analysis focused on functional genes involved in nitrogen fixation as well as 1-aminocyclopropane-1-carboxylic acid (ACC) deaminase, aminopeptidase, and glucanase activities.

Functional profiles of the bacterial communities were clustered into two major groups clearly associated to the wastewater treatment (**Figures 7**, **8**). Cluster 1 included samples treated with HTL-WW after 7, 14, and 21 days (WW_T7, WW_T14, and WW_T21, respectively); whereas cluster 2 grouped untreated samples (C_T0, C_T7, C_T14, and C_T21) as well as treated samples at the beginning of the experiment (WW_T0). Several predicted functions, including endoglucanase (EC:3.2.1.4), leucyl aminopeptidase (EC:3.4.11.1), aminopeptidase N (EC:3.4.11.2), Xaa-Pro aminopeptidase (EC:3.4.11.9), methionyl aminopeptidase (EC:3.4.11.18), D-aminopeptidase (EC:3.4.11.19) (**Figure 7**), as well as nitrogen fixation proteins *Nif*B and *Nif*U, the nitrogen fixation regulation protein CRP/FNR family transcriptional regulator and ACC-deaminase (EC:3.5.99.7) (**Figure 8**), seems to play a discriminating role in the overall system. In particular, from day 7 until the end of the experiment, predicted abundances of genes for aminopeptidase N (EC:3.4.11.2) and endoglucanase (EC:3.2.1.4) increased in the samples treated with HTL-WW (**Figure 7**). In contrast, untreated samples show higher levels of D-aminopeptidase (EC:3.4.11.19) and leucyl aminopeptidase (EC:3.4.11.1) (**Figure 7**).

However, predicted abundances of some enzyme‐encoding nitrogenase genes showed that although *Nif*B and the nitrogen-fixing transcriptional regulator of the CRP/FNR family were present in all rhizosphere samples, their predicted abundance increased in untreated samples from 7 to 21 days (C_T7, CT14 and C_T21; **Figure 8**). Whereas the nitrogen fixation protein *Nif*U remained constant in HTL-WW treated rhizosphere (WW_T0, WW_T7, WW_T14 and WW_T21) and decreased over time in the control samples (C_T7, CT14 and C_T21; **Figure 8**). Finally, the predicted abundance of ACC-deaminase increased in all tobacco rhizosphere samples, especially in HTL-WW treated samples (WW_T7, WW_T14 and WW_T21; **Figure 8**).

## 4 Discussion

The use of wastewater is gaining increasing interest to meet agricultural irrigation needs and to face the increase of population growth and climate change. Although it was reported that wastewater may deliver nutrients and organic matter to the soil (Ungureanu et al., 2020), its composition could have an impact on the microbial communities that contribute to the soil biological fertility, with variable consequences on plant growth and development. In this context, the potential use of hydrothermal liquefaction wastewater (HTL-WW) for irrigation of agricultural crops was explored.

HTL-WW irrigation improved plant chlorophyll content and increased the flower biomass in tobacco plants (**Figure 2** and **Table 2**). These results can be respectively associated with high levels of nitrogen, phosphorus, and potassium as well as macro-nutrients such as sulphates, calcium and magnesium contained in the HTL-WW (**Table 1**) since it is reported their involvement in flowering of different plants (Patel et al., 2006; Erel et al., 2008; Ye et al., 2019) and in chlorophyll content (Wang et al., 2021; Zhang et al., 2021). The tobacco plants treated with HTL-WW have not shown symptoms of heavy metals stress confirming that the concentrations of heavy metals were under harmful levels and anyhow below the official admitted thresholds. The impact of this wastewater on autochthonous microbiota of bulk-soil and rhizosphere was assessed over time using metagenomic approach and bioinformatic tools.

High-throughput sequencing was used to obtain an assessment of the effect of the HTL-WW treatment on resident soil microbial community in the rhizosphere of tobacco. Beta diversity analysis of rhizosphere-associated microbiota demonstrated a shift in the microbial structure especially for bacterial community. In fact, the PCoA exhibited two distinct groups based on water treatments. This behaviour could be due to the different adaptation mechanisms used by several microbial groups to survive and grow under these environmental conditions resulting in a new balance among microbial populations. However, this effect could be temporary. As reported by Frenk et al. (2013) initial variations observed in the soil microbial structure following municipal wastewater irrigation disappeared after three years of treatment suggesting that autochthonous microbiota shown a natural resilience to anthropogenic soil perturbations.

A highly complex bacterial community was also found in the tobacco rhizosphere treated with HTL-WW, in which among the most frequently occurring bacteria were those belonging to Proteobacteria and Actinobacteria. These phyla have been previously reported as dominant populations in the rhizosphere of crops grown in hydroponics and natural environments (Bulgarelli et al., 2013; Sheridan et al., 2017). Several members belonging to the phylum Proteobacteria possess specific genes involved in plant growth-promotion activities (PGP) and can contribute, both by direct and indirect mechanisms, to the development of plants of agricultural interest (Ventorino et al., 2007; Ventorino et al., 2014; Romano et al., 2020b). Although our data did not show significant growth improvements, an increased SPAD levels and flowers number upon treatment with HTL-WW were consistent with possible beneficial effects of Proteobacteria on the overall plant performance. While the relative abundance of Proteobacteria was enough uniform over time in both treated and untreated rhizosphere, the Actinobacteria showed a different trend. In details, the abundance of this phylum remained constant in untreated rhizosphere, whereas it increased over time in HTL-WW treated plants. Members belonging to phylum Actinobacteria are able to synthetize specific hydrolytic enzymes for efficiently decomposition of a wide variety of recalcitrant organic materials as well as to assimilate inorganic nitrogen (Ventorino et al., 2013; Pennacchio et al., 2018; Ventorino et al., 2019). In addition, this microbial group included species that can synthesize plant growth promoting substances and increase nutrient availability and uptake to/by plants as well as exert suppressive effects vs. plant soil-borne pathogens (Chouyia et al., 2020; Fagnano et al., 2020; Di Mola et al., 2021; Chouyia et al., 2022). Once again, the abundance of these bacterial populations was consistent with the high biological fertility potential of treated rhizosphere as demonstrated by a generally enhanced plant performance. Among Actinobacteria, Micrococcaceae and Nocardiaceae were the more abundant bacterial families in the rhizosphere of tobacco HTL-WW treated plants. Micrococcaceae is a well-known stress-tolerant bacterial taxon which has a bioremediation potential as production of bioactive compounds (Caradonia et al., 2019), degradation of phenanthrene, cellobiose and glucose (Li et al., 2019), tolerance to drought and salinity stress (Munoz-Ucros et al., 2021) as well as to heavy metals (Xi et al., 2021). Micrococcaceae is also studied for its plant growth promoting activities such as synthesis of the 1-aminocyclopropane-1-carboxylic acid (ACC) deaminase, indole-3-acetic acid (IAA) and siderophores as well as phosphorus solubilisation even in arid environments (Yang et al., 2019). The high abundance of this taxon in the treated tobacco rhizosphere could be due to the excess of different nitrogen compounds introduced to the soil through HTL-WW or some organic acids (Xi et al., 2021). The use of N by Micrococcaceae, and most likely other bacteria and/or fungi, could explain the missing growth effects on plants treated with HTL-WW, since only a part of its N content was available for plant growth. Moreover, since many species belonging to the Micrococcaceae family are halotolerant, their abundance could have increased because of them ability to tolerate high level of Na^+^ and Cl^-^ present in HTL-WW (Mishra et al., 2018). An increased population of halotolerant bacteria, including also Comamonadaceae family belonging to the Proteobacteria phylum, in the rhizosphere may have had protective effects on plants, a concentration that would be harmful for tobacco and other plants, did not show any stress symptoms. Protective effects of stress tolerant bacteria on plant growth have been documented in tomato and spinach rhizosphere (Ibekwe et al., 2017; Van Oosten et al., 2018).

Nocardiaceae, together Bacillaceae and Pseudomonaceae, are the bacterial taxa present only in the treated rhizosphere. The most abundant family was Nocardiaceae, which play an important ecological role in nature in the recycling of organic matter and recovery oil-contaminated environments as biosurfactant producers. This taxon has also a great potential for plant growth-promotion potential and biocontrol of different plant diseases (Yang et al., 2019). Nocardiaceae are recently detected in the rhizosphere of different crops as well as in activated sludge, wastewater, digestate and hydrocarbon polluted soils (Ali et al., 2021).

Bacillaceae and Pseudomonaceae were also present mainly in the rhizosphere of plants treated with HTL-WW, although their abundance decreased over time. Members belonging to these bacterial families include many rhizobacteria which are able to promote plant growth through various mechanisms, including phosphate solubilization and synthesis of phytohormones (Shrivastava et al., 2018), as well as to exert a biocontrol action against various soil borne plant pathogens (Beneduzi et al., 2012). Furthermore, many species belonging to the Bacillaceae family could degrade cellulose, hemicellulose, pectin, and lignin (Di Pasqua et al., 2014), suggesting their possible involvement in the degradation and mineralization of organic and humic compounds in the soil.

According to Lüneberg et al. (2019) Ascomycota and Basidiomycota dominated in most agricultural soil. These phyla constituted the key primary decomposers of soil organic matter, and they are among drivers in regulating the ecosystem functions. Most members belonging to these phyla are saprotrophic fungi that participated in critical processes, such as the decomposition and mineralization of recalcitrant and labile compounds as well as xenobiotic substances (Yang et al., 2022). Ascomycota, previously described as sensitive to high nitrogen, phosphorous and carbon inputs (Chen et al., 2017), did not seem to be affected by the higher nutrient levels of HTL-WW vs. control water. Within this phylum, Nectriaceae was the most abundant family in treated samples. This result was consistent with those of Lüneberg et al. (2019), who also reported this fungal taxon as the most abundant in a field irrigated with untreated wastewater. The Nectriaceae family has also a key role in the nitrogen bioremediation (Cheng et al., 2020) in N-rich environments, such as the soil irrigated with HTL-WW. The fungal families selected only in treated tobacco rhizosphere were Saccharomycetales-incertae-sedis and Trichocomaceae, belonged to phyla of Ascomycota and Basidiomycota respectively. Furthermore, Saccharomycetales-incertae-sedis and Trichocomaceae are both involved in bioremediation of heavy metals, especially cadmium, lead, chromium, nickel, copper, and manganese (Malikula et al., 2022). Thus, as mentioned before, these fungal families may have protected the plants by degrading the heavy metals introduced with HTL-WW.

The study of the potential bacterial activities is fundamental to establish new agricultural practices as the use of HTL-WW for irrigation purposes. In this context, differences in bacterial functioning between HTL-WW treated and non-treated tobacco rhizosphere were investigated based on predicted abundances of enzyme-encoding genes involved in plant growth promotion, soil fertility and suppression disease such as nitrogen fixation, ACC deaminase, aminopeptidase, and glucanase (Hong and Meng, 2003; Fiorentino et al., 2016; Norman et al., 2020; Orozco-Mosqueda et al., 2020; Romano et al., 2020b). The clustering of predicted functions demonstrated that potential gene abundances were strongly affected by water treatment since two major clusters were observed clearly associated to the HTL-WW treatment. Shift in microbial community structure was also previously observed when different wastewaters, as household, industrial, or olive mill, were used for plant irrigation (Zhao et al., 2014; Giagnoni et al., 2016; Krause et al., 2020). Changes in the rhizosphere microbial community could be due to modifications of root exudates resulting from the high amount of nutrients and the different nitrogen forms introduced by wastewater as well as by the C/N ratio. The shift in microbial community structure could also be attributed to the high availability and quality of the carbon sources supplied by wastewater irrigation. Indeed, following the use of carbon-rich wastewater as HTL-WW, the presence of easily degradable carbon compounds could stimulate the growth of copiotrophic as well as of cellulolytic microorganisms (Marschner et al., 2003), as also confirmed by the increasing of predicted abundance of endoglucanases following wastewater treatment. In addition, bacterial endoglucanases confer a competitive advantage against soil-borne plant pathogens and therefore, their presence could be considered as a mechanism to improve plant resistance to biotic stress (Hong and Meng, 2003). HTL-WW irrigation increased also the predicted abundance of ACC-deaminase; an enzyme produced by some plant growth-promoting microbes to alleviate negative effects due to ethylene production. Synthesis of ACC deaminase could also improve plant tolerance to several abiotic or biotic stress, such as drought, salinity, and high temperature (del Carmen Orozco-Mosqueda et al., 2020; Romano et al., 2020b).

The predicted abundances of genes encoding aminopeptidase N and nitrogen fixation protein NifU increased or remained constant in HTL-WW treated samples. These predicted bacterial functions could help to increase soil fertility and enhance plant growth, since aminopeptidases producing bacteria can degrade proteins from soil organic matter increasing amino acid availability in the near-cell environment (Norman et al., 2020), as well as nitrogen-fixers can supply plants with this essential nutrient and improving soil fertility (Fiorentino et al., 2016).

## 5 Conclusion

Although the use of industrial wastewater to satisfy crop water requirements could have several economic and environmental advantages, the response of the soil microbiota to different wastewater sources must be ascertain on a case-by-case basis, since this may critically affect the overall soil biological fertility. This work improves the knowledge about the responses of indigenous microbial populations to anthropogenic activities, particularly related to the potential ecological risks of using untreated industrial wastewater derived from hydrothermal liquefaction process (HTL-WW) for irrigation purposes and the capacity of natural ecosystems to develop an adapted microbiota, ultimately leading to the establishment of a new microbial community affecting the soil fertility properties.

Based on the results obtained in this study, the wastewater from the hydrothermal liquefaction of organic wastes could be an important source of irrigation water which could contribute to reduce the increasing pressure on freshwater resources. However, further specific investigations will be necessary to assess the long-term impact of this untreated industrial wastewater on soil biological fertility and possible critical effects on the biogeochemical cycles of relevant soil nutrients.

## Data availability statement

The datasets presented in this study can be found in online repositories. The names of the repository/repositories and accession number(s) can be found below: https://www.ncbi.nlm.nih.gov/, PRJNA939137.

## Author contributions

WG carried out the experiments and drafted the manuscript. VC performed agronomic analyses and analysed the results for the part. AM reviewed and edited the manuscript. IR performed functional prediction analysis and drafted the manuscript for this part. VV reviewed and edited the manuscript, conceived the concepts of the study and participated in its design. OP reviewed the manuscript and contributed to its conceiving. All authors have read and approved the final manuscript.
